# Performance improvement of an X-ray ionization beam position monitor for soft X-ray free-electron lasers

**DOI:** 10.1107/S1600577526005382

**Published:** 2026-06-18

**Authors:** SunMin Hwang, Seonghan Kim, Woojin Song, Seungcheol Lee, Garam Hahn, Hoyoung Jang, Minzy Jeong, Gyeongbo Kang, Sanghan Park, HyoJung Hyun

**Affiliations:** a Pohang Accelerator Laboratory, POSTECH, Pohang, Gyeongbuk37673, Republic of Korea; Deutsches Elektronen-Synchrotron, Germany

**Keywords:** X-ray ionization beam position monitor, soft X-ray free-electron laser, beam position diagnostics, gas-based detector

## Abstract

An X-ray ionization beam position monitor (XIBPM) has been developed and evaluated for beam position diagnostics at a soft X-ray free-electron laser (FEL). The redesign was guided by electrostatic simulations and focused on mitigating electric-field distortions near the microchannel plate (MCP) and improving the internal electrode geometry and structural stability. The introduction of a conductive shielding cover effectively suppressed electric-field distortion from the MCP lead, substantially reducing displacement errors and improving measurement stability. A mesh-free electrode structure was also investigated, reducing photo-ion loss and increasing photo-ion transmission; however, under high-intensity FEL operation, the enhanced signal amplitude occasionally led to saturation effects. Beam position measurements were performed at photon energies of 500–1100 eV in both pink-beam and mono-beam modes. Under optimized conditions, a beam position resolution of approximately 24–25 µm was achieved for pulse-by-pulse analysis and improved to approximately 10–12 µm when averaging over ten pulses. The transverse ion profiles were found to be significantly broader than the actual photon beam size, reflecting intrinsic ion-transport dynamics and detector point-spread effects that dominate over the intrinsic photon beam size. These results demonstrate that the shielding cover represents the most significant improvement for accurate and stable beam position monitoring, while the electrode configuration can be selected flexibly depending on the operating regime. The improved XIBPM provides a practical, non-invasive solution for beam position diagnostics in soft X-ray FEL beamlines.

## Abstract 2

The performance evaluation and improvement of an X-ray ionization beam position monitor for soft X-ray free-electron lasers is presented.

## 1. Introduction

X-ray free-electron lasers (XFELs) provide intense, ultrashort and highly coherent X-ray pulses, enabling a wide range of time-resolved and nonlinear experiments. However, owing to the self-amplified spontaneous emission (SASE) process (Kondratenko & Saldin, 1980 ▸; Bonifacio *et al.*, 1984 ▸), XFEL radiation exhibits significant pulse-to-pulse fluctuations in intensity, spectral distribution, central photon energy and beam position (Saldin *et al.*, 1998 ▸; Saldin *et al.*, 2010 ▸). Reliable, non-invasive photon diagnostics are therefore indispensable for quantitative data analysis and stable beamline operation.

At hard X-ray beamlines, beam position monitoring is commonly realized using solid-state-based devices such as quadrant beam position monitors (QBPMs), which detect scattered X-rays from thin foils (Alkire *et al.*, 2000 ▸; Feng *et al.*, 2011 ▸; Tono *et al.*, 2011 ▸). In the soft X-ray regime, however, strong absorption in solid materials severely limits the applicability of such approaches. As a result, gas-based diagnostics exploiting photo-ionization processes (Tiedtke *et al.*, 2008 ▸; Tiedtke *et al.*, 2014 ▸) have been widely adopted, most notably gas monitor detectors (GMDs) for pulse energy monitoring and normalization. While some GMD systems can provide averaged beam position information (Sorokin *et al.*, 2019 ▸), they are not designed to deliver direct, spatially resolved measurements of the transverse beam profile. In the soft X-ray beamline of the Pohang Accelerator Laboratory X-ray Free-Electron Laser (PAL-XFEL) (Ko *et al.*, 2017 ▸; Kang *et al.*, 2017 ▸), the GMD is used exclusively as an intensity monitor and does not provide transverse beam position information.

To overcome this limitation, an X-ray ionization beam position monitor (XIBPM) (Kim *et al.*, 2024 ▸) was previously developed for the soft X-ray beamline of the PAL-XFEL. The XIBPM visualizes beam position changes through two-dimensional distributions of photo-ions generated in residual gas under ultra-high-vacuum conditions (Sachwitz *et al.*, 2008 ▸; Mießner *et al.*, 2011 ▸) or injected gas and detected by a microchannel plate (MCP) coupled to a phosphor screen. Operation based on residual gas provides a simple and compact solution without the need for additional gas handling systems, although injected gas can be used to enhance the signal level. Initial beam tests demonstrated the feasibility of relative beam position monitoring in both pink-beam and mono-beam modes, with an improved signal-to-noise ratio achieved through krypton gas injection.

Despite these encouraging results, subsequent studies revealed several non-ideal behaviors that limited measurement accuracy and robustness. In particular, the measured ion distributions exhibited unexpected profile broadening and voltage-dependent peak shifts, and the extracted beam widths were significantly larger than those measured by independent diagnostics. These effects were traced to distortions in the internal electric field, especially in the vicinity of the MCP high-voltage lead, which were not fully accounted for in the original detector design.

Motivated by these observations, the present work aims to systematically optimize the performance of the XIBPM through simulation-guided internal structure redesign. Electrostatic field-map analyses and particle-tracking simulations were employed to identify the dominant sources of electric-field non-uniformity. Based on these results, key design modifications – including the introduction of an MCP shielding cover, removal of mesh electrodes, and revision of the electrode and support structures – were implemented. The redesigned XIBPM was subsequently evaluated through two dedicated machine-study campaigns, with quantitative comparisons with downstream reference diagnostics under a wide range of operating conditions.

## 2. Overview of the XIBPM

The XIBPM developed for the PAL-XFEL soft X-ray beamline (Park *et al.*, 2018 ▸; Jang *et al.*, 2020 ▸) is based on the detection of photo-ions generated by the interaction between the X-ray beam and residual or injected gas inside the vacuum chamber. The ions are accelerated by an applied electric field and detected using an MCP coupled to a phosphor screen (F2223-21P with P43, Hamamatsu Photonics) and an external CCD camera (Manta G-046B, Allied Vision), allowing direct visualization of beam position changes as two-dimensional ion distributions.

In the initial implementation, the electric field was generated using a repeller plate and two tungsten mesh electrodes designed to approximate an equipotential region between the ionization volume and the MCP. While beam tests demonstrated the feasibility of beam position monitoring, detailed analysis revealed limitations including excessive profile broadening, voltage-dependent peak shifts, and sensitivity to internal electric-field distortions. In particular, the influence of the MCP high-voltage lead was identified as a critical source of field non-uniformity.

These observations motivated a comprehensive redesign of the internal structure, supported by electrostatic simulations and particle-tracking studies, as described in the following section.

## 3. Simulation-driven internal structure redesign

The initial performance evaluation of the XIBPM revealed that the measured beam profiles and position stability were strongly influenced by the internal electric-field configuration. In particular, unexpected broadening of the ion distribution and systematic shifts of the measured beam position were observed, depending on the applied voltages and MCP operating conditions. These behaviors suggested the presence of local electric-field distortions inside the detector and motivated a detailed investigation of the internal structure.

To identify the dominant sources of electric-field non-uniformity, electrostatic field-map calculations and multi-particle tracking simulations were performed using the *CST Studio Suite*, a three-dimensional finite-element-based simulation tool (CST Studio Suite, 2026 ▸). An idealized reference model was first constructed, assuming perfect mechanical alignment, flat tungsten mesh electrodes, the absence of an MCP high-voltage lead, and an approximately equipotential field generated by properly biased electrodes. Deviations from this ideal case were then systematically introduced to evaluate their impact on the electric-field distribution and ion trajectories.

The investigated non-ideal conditions included floating or incorrectly biased mesh electrodes, slight mechanical mis­alignment of the internal structure, curvature of the tungsten mesh, and the presence of the MCP high-voltage lead, as implemented in the actual detector. The electrostatic simulations showed that neither mesh curvature nor incorrect mesh biasing alone produced significant deviations from the ideal field distribution. Mechanical misalignment resulted in only minor perturbations in the static field map. In contrast, the presence of the MCP high-voltage lead introduced a pronounced local electric-field distortion near the detector plane.

To quantify the impact of these distortions on ion transport, particle tracking simulations were performed assuming a cylindrical source of stationary Kr^1+^ ions with a radius of 1 µm. Ion trajectories were calculated in the *X–Z* plane, and their spatial distributions on the MCP surface were analyzed. While most non-ideal cases yielded trajectories comparable with those of the ideal configuration, the misaligned structure and, more prominently, the MCP high-voltage lead produced abnormal trajectory patterns. The relative centroid deviations derived from the projected trajectories reached approximately 8.2% for the MCP high-voltage lead and 7.9% for the mis­alignment case.

**Figure 1:**
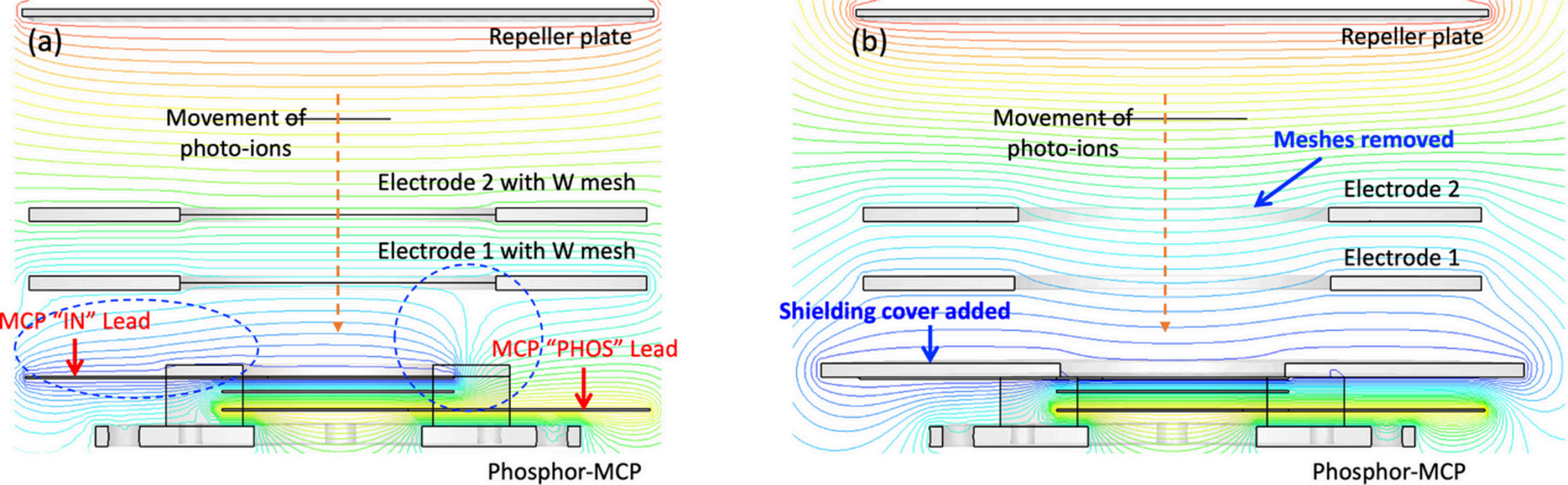
Electrostatic field-map simulations of the XIBPM. (*a*) Original design showing strong local electric-field distortion caused by the MCP high-voltage lead. (*b*) Redesigned configuration with a conductive shielding cover biased at the MCP potential, effectively suppressing the field distortion and providing more uniform electric field near the detector.

Fig. 1 ▸(*a*) shows the simulated equipotential lines for the original XIBPM design. A strong local distortion of the electric field is observed near the MCP lead, where the high-voltage connection introduces a localized perturbation. This distortion deflects photo-ion trajectories toward the detector plane, resulting in an apparent shift of the beam centroid and increased sensitivity to voltage settings.

Based on these results, a conductive shielding cover was introduced above the MCP and biased at the same potential as the MCP input. As shown in Fig. 1 ▸(*b*), the shielding cover effectively suppresses the electric-field distortion caused by the MCP lead, resulting in a significantly more uniform field distribution near the detector plane.

In parallel with the detector-side modification, the mechanical support structure was redesigned to mitigate alignment-related errors identified in the simulations. The original four-post support configuration was replaced by a one-piece block structure to improve mechanical rigidity and alignment reproducibility between the MCP and the electrode assembly.

Following this structural improvement, the electric-field creation side of the XIBPM was further redesigned. In the original design, the tungsten mesh electrodes were not self-supporting and therefore required a sandwich structure, in which the mesh was held between two ring-type electrodes to form each electrode assembly. In the redesigned configuration, the mesh was removed and replaced by self-supporting ring-type electrodes with slightly different inner diameters, providing controlled field shaping. This modification eliminates the need for the sandwich structure, simplifies the internal assembly, and improves mechanical robustness and alignment reproducibility. In addition, the removal of the mesh reduces ion loss and potential scattering-induced background without compromising the effective ionization region.

**Figure 2:**
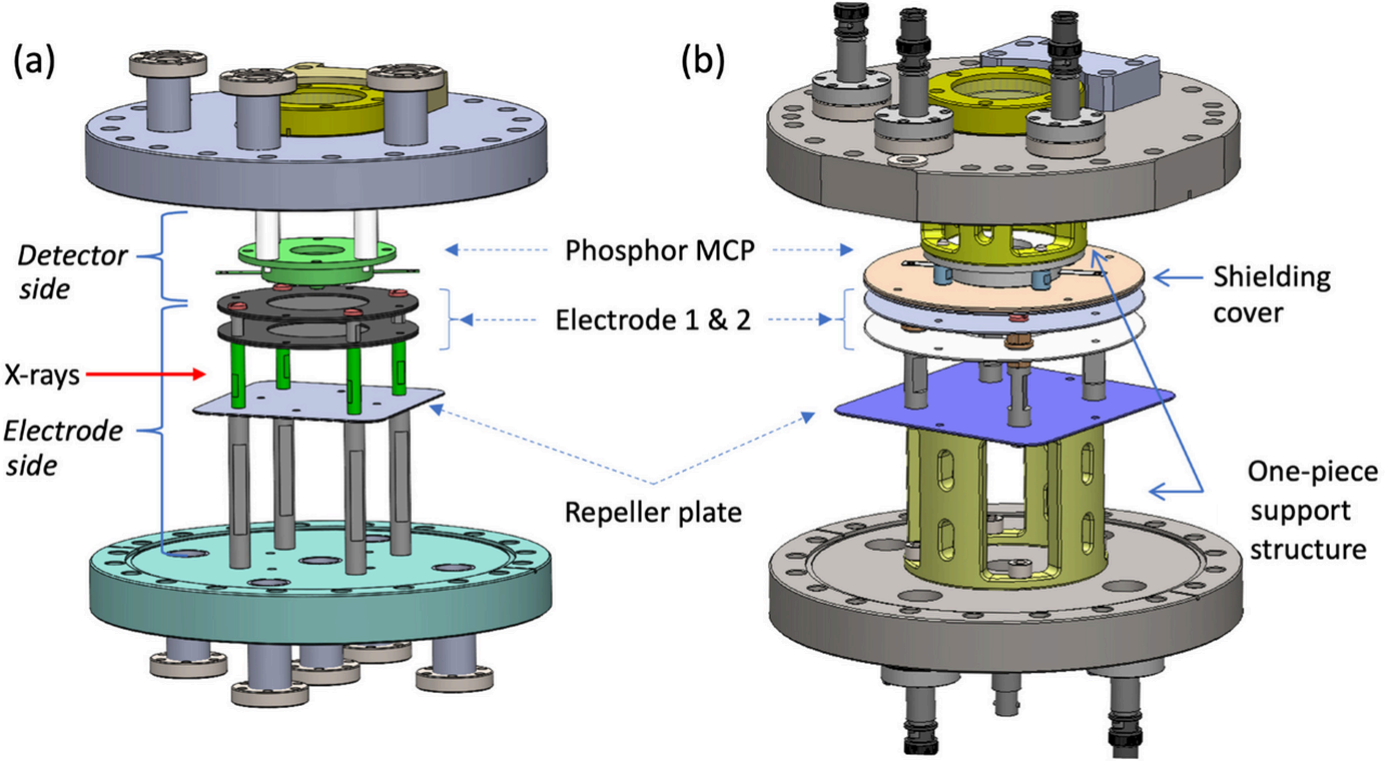
Schematic comparison of the (*a*) original and (*b*) redesigned XIBPM structures. The redesign includes the introduction of a shielding cover on the detector side, removal of mesh electrodes, modified electrode geometry on the electrode side, and replacement of the multi-post support structure with a one-piece block.

A schematic comparison of the original and redesigned XIBPM structures is presented in Fig. 2 ▸. The figure highlights the separation between the detector side and the electric-field creation side, as well as the key design changes introduced in this work. The redesign was implemented without altering the overall footprint or external interfaces of the device and therefore did not require modification of the existing installation conditions.

The simulation-guided redesign described in this section forms the basis for the performance improvements evaluated in the following sections. In particular, the introduction of the MCP shielding cover and the modified electrode geometry were expected to mitigate electric-field-induced artifacts and enhance the robustness of beam position measurements over a wide range of operating conditions. These design modifications were progressively implemented and experimentally evaluated during two dedicated machine-study campaigns conducted in January and May 2025.

## 4. Performance evaluation

This section presents a comprehensive performance evaluation of the XIBPM after the internal structure redesign. The assessment is based on two dedicated machine-study campaigns conducted in January and May 2025 at the PAL-XFEL soft X-ray beamline. The performance of the improved XIBPM is examined through reference beam size measurements, quantitative comparisons of beam position displacements with independent diagnostics under various operating conditions, and profile characterization.

### 4.1. Experimental conditions and procedure

All measurements were carried out at the PAL-XFEL soft X-ray beamline using photon energies of 500, 700, 900 and 1100 eV. The XIBPM was operated exclusively in krypton gas mode throughout the study to ensure sufficient signal-to-noise ratio, while operation using residual gas alone was not considered. During krypton gas injection, the chamber pressure increased from a base pressure of ∼1 × 10^−7^ Torr to ∼1 × 10^−6^ Torr in the January 2025 campaign and ∼1 × 10^−5^ Torr in the May campaign. Correspondingly, the krypton partial pressure measured using a residual gas analyzer (RGA) increased from ∼2 × 10^−10^ Torr to ∼8  × 10^−8^ Torr and ∼2  × 10^−7^ Torr, respectively.

Beam position changes were intentionally introduced by adjusting upstream mirror motors. Vertical displacements were generated by scanning the M3 mirror, whereas horizontal displacements were produced by scanning the M1 mirror. In addition to the mirror scans, the influence of the internal electric-field configuration was systematically investigated by varying the repeller voltage and the MCP gain voltage under selected operating conditions. For the original voltage configuration, the voltages applied to electrodes 1 and 2 were determined based on their geometric distances to approximate an equipotential condition.

Two dedicated machine-study campaigns were conducted with distinct objectives. The January 2025 campaign focused on evaluating the effect of the shielding cover installed above the MCP. Measurements were performed in both vertical and horizontal directions under pink-beam operation to assess the impact of suppressing electric-field distortion caused by the MCP lead on beam position stability and accuracy. The May 2025 campaign extended the study to include the redesigned electrode structure and additional voltage configurations. In this campaign, detailed performance evaluations were carried out primarily in the horizontal direction, reflecting the intended operational configuration in which the XIBPM will be installed downstream of the vertical exit slit for routine beamline operation. Measurements were performed under both pink-beam and mono-beam modes, enabling direct comparison of detector performance across different beam modes as well as an investigation of the effects of voltage configurations.

**Figure 3:**
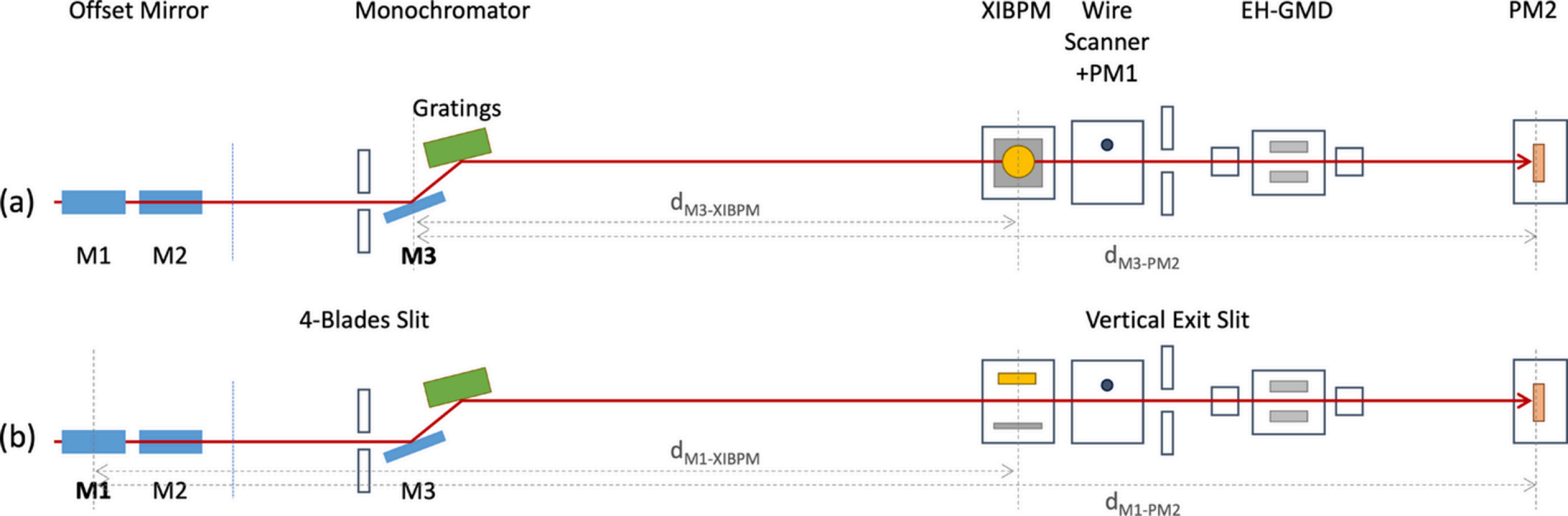
Experimental configurations for beam position measurements in a side view. (*a*) Vertical configuration using an M3 mirror scan and (*b*) horizontal configuration using M1 mirror scan. The known geometrical distances between the mirror, XIBPM and PM2 define the reference for quantitative evaluation of relative beam displacement. The XIBPM was positioned downstream of the monochromator and upstream of the wire scanner, with a 90° rotation applied depending on the measurement configuration.

For all displacement measurements, the XIBPM data were compared with those obtained from a downstream pneumatic profile monitor equipped with a YAG:Ce screen (PM2). The experimental configurations used for the vertical and horizontal beam position measurements are illustrated in Fig. 3 ▸. The XIBPM was installed downstream of the monochromator and upstream of the wire scanner and PM2, and the measurement direction was selected by applying the appropriate mirror scan and detector orientation. The known distances between the mirror, XIBPM and PM2 define a fixed geometrical relationship, which forms the basis for the quantitative evaluation of relative beam displacement errors discussed in the following sections. For each mirror position, typically 300–600 shots were recorded depending on the campaign, and the analysis was performed using pulse-to-pulse data as well as data averaged over ten pulses.

The spatial calibrations of both imaging systems were determined using grid-pattern images recorded under the same CCD camera settings used during beam measurements. The resulting pixel-to-length conversion factors were determined to be 29.4 ± 0.4 µm per pixel for the XIBPM and 66.4 µm per pixel for the PM2, and these calibration factors were consistently applied in converting pixel-based beam positions and profile widths into physical units throughout the analysis.

**Figure 4:**
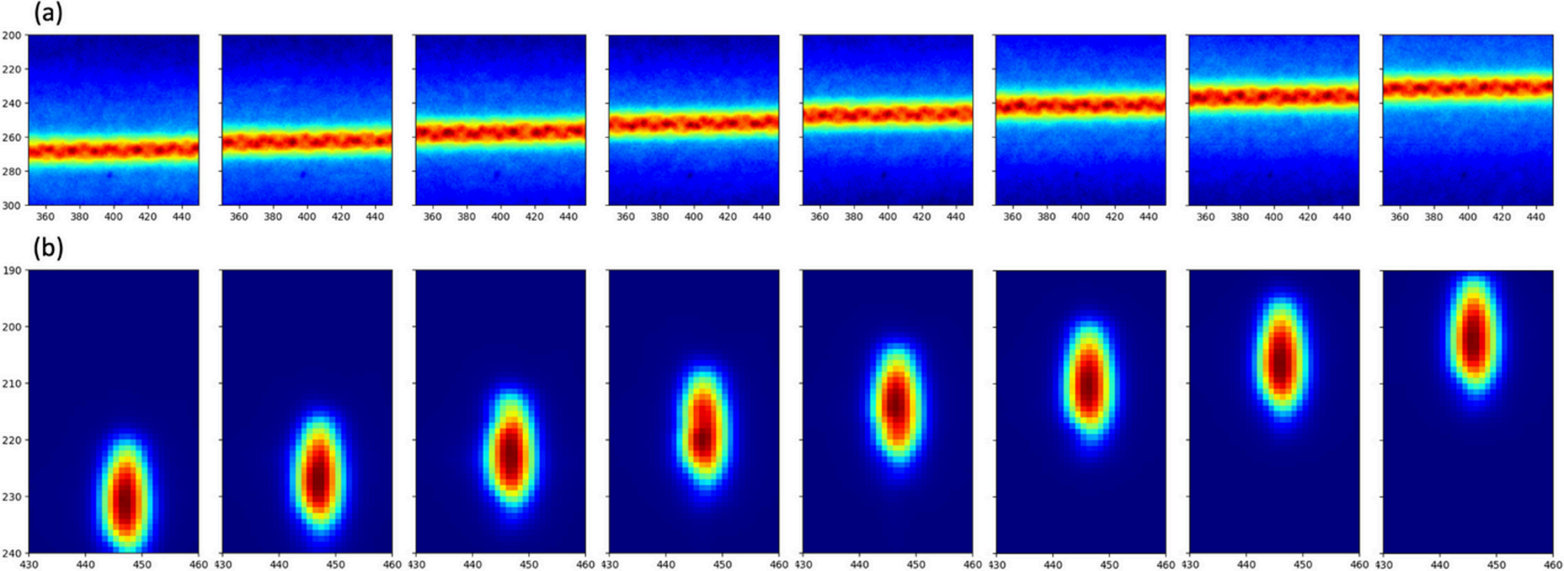
Representative two-dimensional images recorded during a vertical displacement scan at 900 eV in pink-beam mode. (*a*) Photo-ion images measured by the XIBPM and (*b*) corresponding beam images obtained from the PM2. The measurements were performed with a repeller voltage of +3000 V and an MCP gain voltage of −1600 V, with the shielding cover applied. The image sequences demonstrate the systematic displacement of the beam position induced by mirror motion.

Representative examples of the two-dimensional images recorded during a vertical displacement scan are shown in Fig. 4 ▸. The XIBPM photo-ion images are presented in Fig. 4 ▸(*a*), while the corresponding reference beam images obtained from the PM2 are shown in Fig. 4 ▸(*b*). The data were acquired at 900 eV in pink-beam mode with a repeller voltage of +3000 V and an MCP gain voltage of −1600 V, with the shielding cover applied. The image sequences illustrate the continuous and stable shift of the beam position induced by mirror motion.

### 4.2. Reference beam size measurements

Independent vertical beam size measurements were conducted in pink-beam mode using a tungsten wire scanner and charge readout from the downstream gas monitor detector (EH-GMD). A tungsten wire with a diameter of 500 µm was employed for all scans, and the corresponding photo-electron and photo-ion signals were recorded by the EH-GMD with 30 pulses accumulated at each motor position.

**Figure 5:**
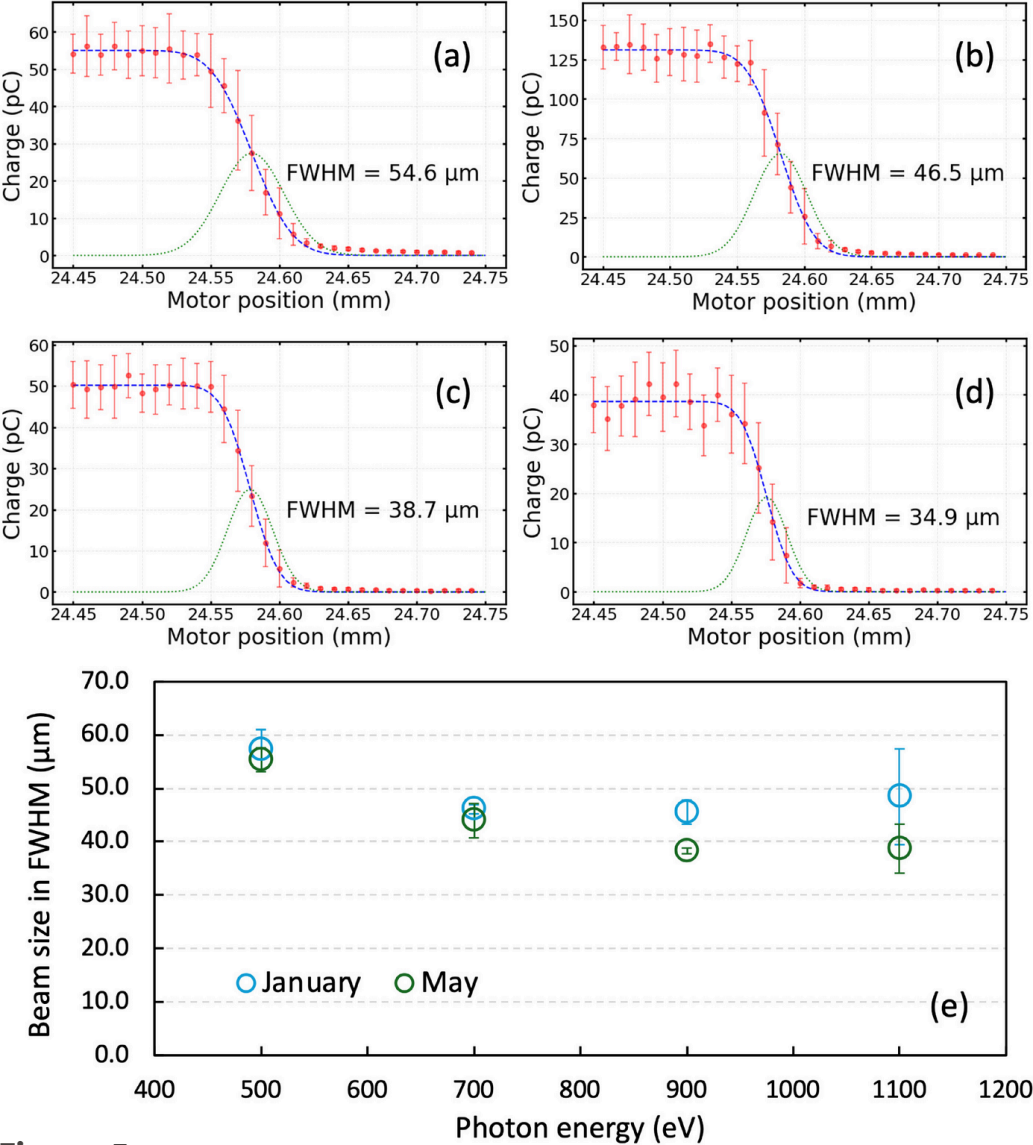
(*a*)–(*d*) Representative wire scanner vertical beam size measurements at photon energies of 500, 700, 900 and 1100 eV, respectively. The photo-electron charge recorded by the EH-GMD is shown as a function of tungsten wire position (500 µm diameter), and the solid curves represent fits used to extract the FWHM. (*e*) Statistical summary of vertical beam sizes measured during the January and May 2025 machine-study campaigns. Beam sizes extracted from both photo-electron and photo-ion charge signals were combined. Symbols represent mean FWHM values, and error bars indicate the corresponding standard deviations for each photon energy.

Figs. 5 ▸(*a*)–5(*d*) present representative examples of the beam size measurements obtained at photon energies of 500, 700, 900 and 1100 eV, respectively. In each case, the photo-electron charge measured by the EH-GMD was recorded as a function of the wire position and fitted to determine the beam size. The extracted full width at half-maximum (FWHM) values are indicated in the respective panels and demonstrate a systematic reduction of the vertical beam size with increasing photon energy.

The FWHM decreases from approximately 55 µm at 500 eV to approximately 35 µm at 1100 eV. The fitting procedure remained stable over the investigated photon energy range, and the associated statistical uncertainties are small compared with the observed energy-dependent trend.

Fig. 5 ▸(*e*) summarizes all vertical beam size measurements obtained during the January and May 2025 machine-study campaigns. Beam sizes extracted from both photo-electron and photo-ion charge signals were combined, and the mean values together with their corresponding standard deviations were calculated for each photon energy. The results from the two campaigns show generally consistent trends, with the exception of a noticeable deviation at 900 eV that exceeds the estimated measurement uncertainty. Overall, the measurements indicate stable intrinsic beam size and beamline optics conditions over the measurement period, and the remaining differences are attributed to variations in machine conditions and measurement statistics.

These reference measurements provide a reliable benchmark for comparison with the XIBPM profiles discussed in the following section.

### 4.3. Results and discussion

#### 4.3.1. Effect of the shielding cover

The effect of the shielding cover installed above the MCP was primarily evaluated during the January 2025 machine-study campaign under pink-beam operation. Beam position measurements were performed in both vertical and horizontal configurations to assess the impact of suppressing electric-field distortion originating from the MCP high-voltage lead.

**Figure 6:**
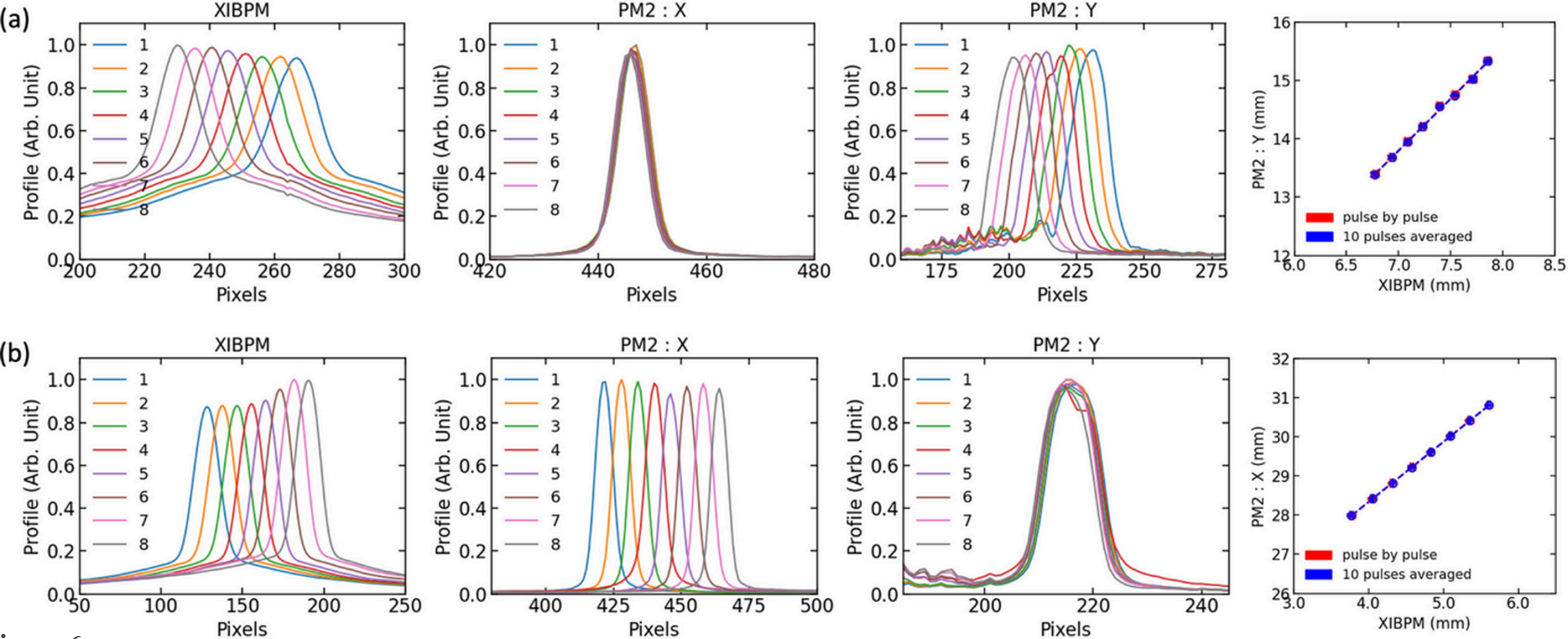
Representative examples illustrating the displacement analysis procedure for the XIBPM. (*a*) Vertical displacement configuration and (*b*) horizontal displacement configuration measured at 900 eV in pink-beam mode with a repeller voltage of +3000 V and an MCP gain voltage of −1600 V under the shielding cover applied. The beam positions were extracted from the XIBPM and PM2 profiles and compared through linear fitting. Consistent correlations are observed for pulse-by-pulse data and data averaged over ten pulses. The slopes obtained from the linear fits are compared with the expected geometrical distance ratios to derive the relative displacement error.

Figs. 6 ▸(*a*) and 6(*b*) present representative results obtained in the vertical and horizontal configurations, respectively, with the shielding cover applied. The XIBPM and PM2 profiles shift systematically as the upstream mirror is scanned. A clear linear correlation between the beam positions measured by the XIBPM and those obtained from the PM2 is observed. The extracted slopes remain consistent across different analysis methods, including pulse-by-pulse evaluation and averaging over ten pulses. On the basis of this consistency, the quantitative results discussed below are derived from the data averaged over ten pulses.

**Figure 7:**
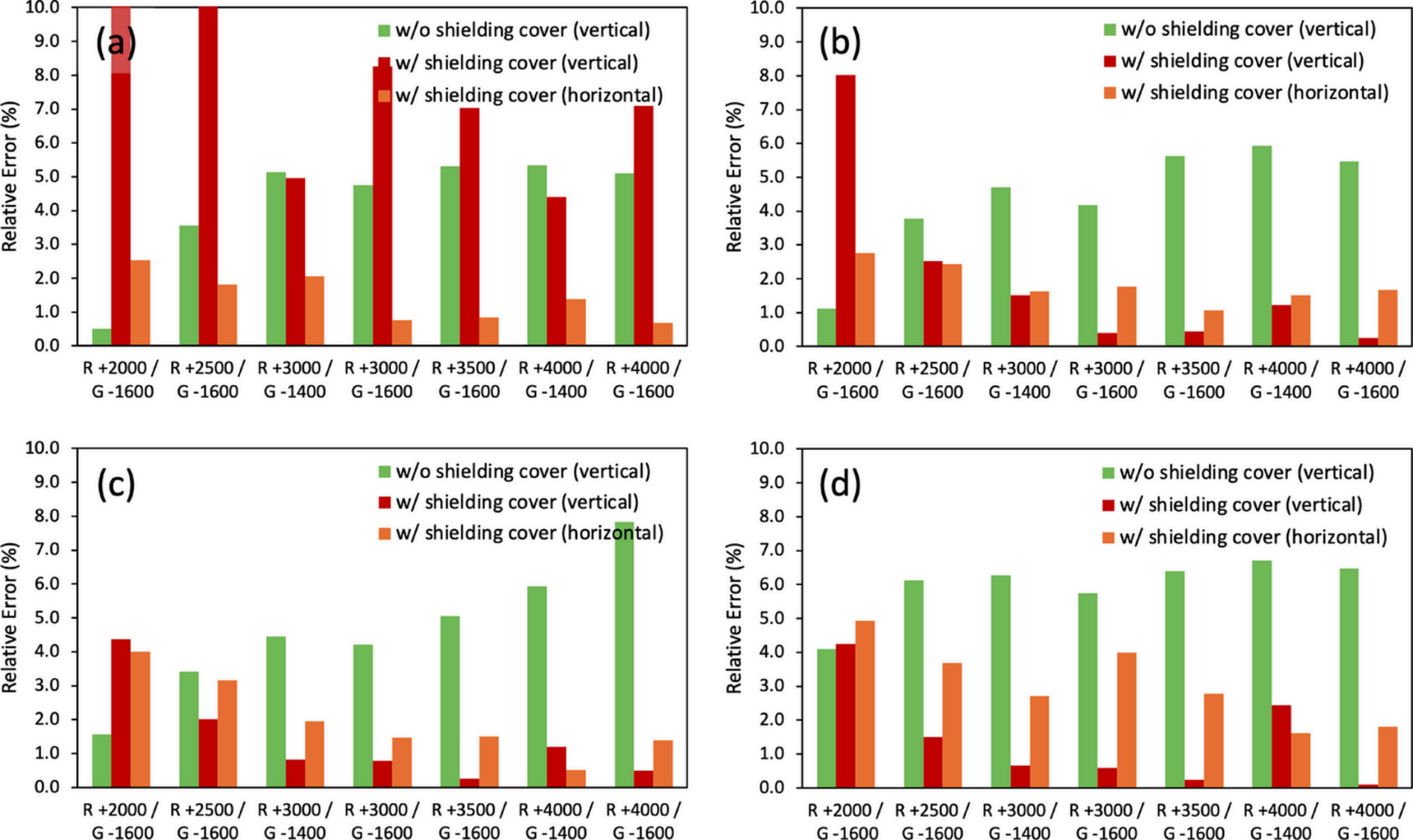
Relative percentage displacement errors measured in pink-beam mode at photon energies of (*a*) 500 eV, (*b*) 700 eV, (*c*) 900 eV and (*d*) 1100 eV as a function of the voltages for the repeller plate (R) and the MCP gain (G) of the XIBPM. The shielding cover generally improves the robustness of the displacement measurement, although the reduction in displacement error is not monotonic for every individual voltage setting. The results are based on the average of every ten pulses.

The relative displacement errors observed in pink-beam mode at photon energies of 500, 700, 900 and 1100 eV, as a function of the repeller voltage and MCP gain, are summarized in Fig. 7 ▸. In general, the application of the shielding cover resulted in reduced displacement errors compared with the unshielded configuration. This behavior is consistent with suppression of trajectory distortion associated with the MCP high-voltage lead, as suggested by the electrostatic simulations.

At 500 eV, the relative displacement error in the vertical configuration remained noticeably larger than at higher photon energies, ranging from approximately 4% to 14%. This increase was strongly influenced by the condition of the reference PM2 YAG:Ce screen. During the vertical measurements, the X-ray beam partially overlapped with a damaged region of the YAG screen, leading to a slight deviation of the PM2 profile from an ideal Gaussian shape. As the beam size increases at lower photon energies, this distortion had a more pronounced impact at 500 eV, resulting in an apparent increase in the displacement error.

From a signal-to-noise perspective, larger statistical errors would be expected at higher photon energies, particularly at 1100 eV where the photon flux and detector response are reduced. However, the comparable error levels observed at 900 eV and 1100 eV indicate that the dominant contribution to the vertical displacement error originated from systematic distortion in the reference PM2 measurement rather than from statistical noise in the XIBPM signal.

In contrast, the horizontal configuration exhibited consistently smaller relative displacement errors, typically below 5% across the investigated photon energy range when the shielding cover was applied. At 500 eV, the relative error was reduced to below approximately 3%, in clear contrast to the vertical case. For the horizontal measurements, the beam position on the PM2 YAG screen was intentionally adjusted to avoid the damaged region, resulting in well defined Gaussian reference profiles. Under these conditions, the observed energy dependence of the displacement error followed the expected statistical trend, with a slight increase observed at 1100 eV attributable to reduced signal-to-noise ratio.

Taken together, these results indicate that the shielding cover reduces electric-field-induced artifacts in the XIBPM and improves beam position measurement reliability. At the same time, they demonstrate that the apparent photon-energy dependence of displacement error can be significantly influenced by limitations of the reference diagnostic, underscoring the importance of reference-detector integrity in beam position monitor evaluations.

#### 4.3.2. Effect of redesigned electrode structure and voltage configuration

The influence of the redesigned electrode structure and voltage configuration on beam position measurements was investigated during the May 2025 machine-study campaign. Following the implementation of the mesh-free electrode geometry, three representative voltage settings were examined: the original voltage configuration (Set A), a configuration with electrodes 1 and 2 left floating (Set B), and configurations derived from electrostatic simulations with a magnification close to unity (Set C). Measurements were performed primarily in the horizontal direction, corresponding to the intended operational configuration in which the XIBPM will be installed downstream of the vertical exit slit for routine beamline use.

In pink-beam mode, quantitative interpretation of the displacement error was constrained by saturation effects in the XIBPM signal. After removal of the mesh electrodes, saturation-like behavior was observed in most voltage configurations over the investigated photon-energy range, except at 1100 eV. Consequently, the relative displacement errors obtained under pink-beam conditions are not considered sufficiently reliable for a detailed comparison of voltage configurations.

**Figure 8:**
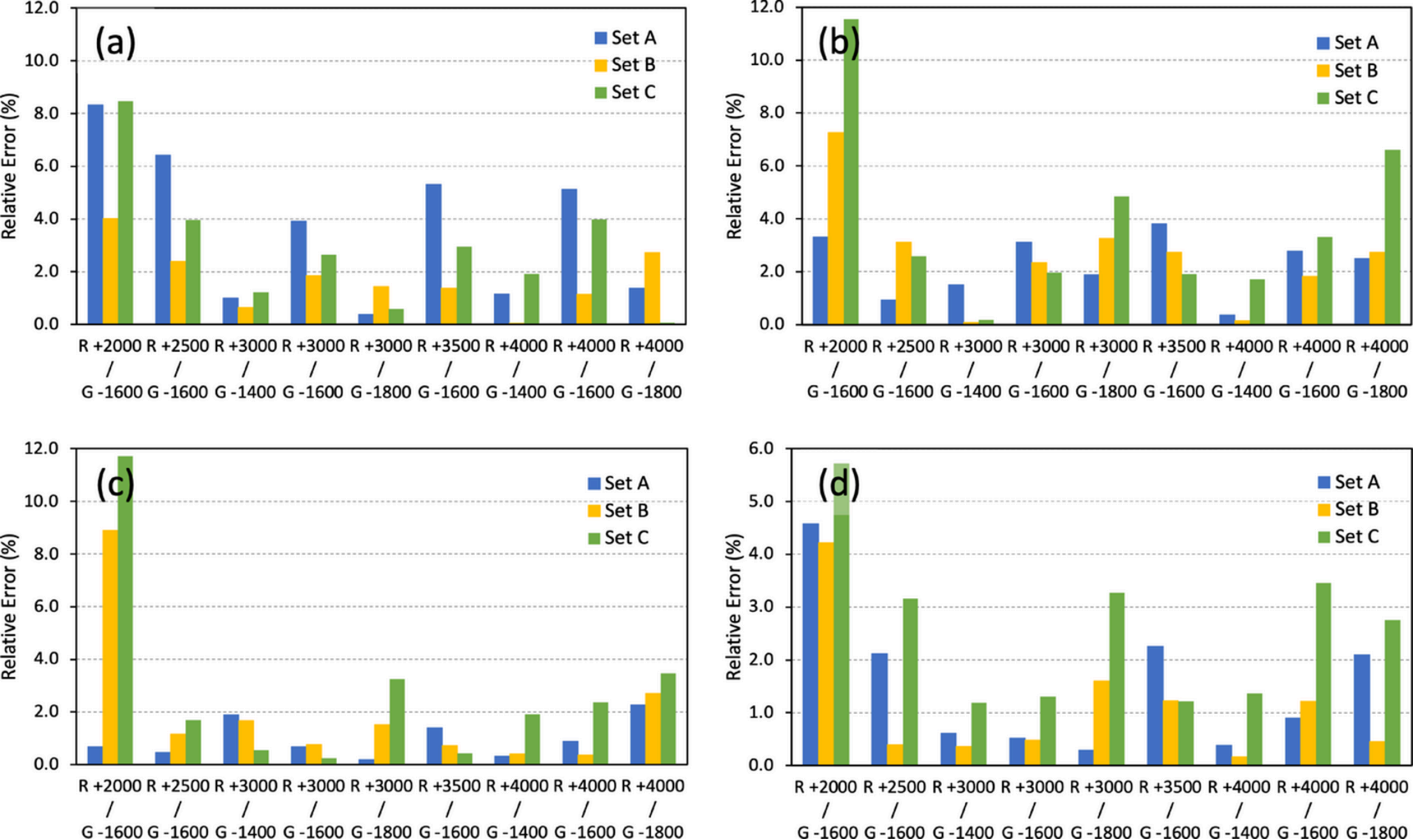
Relative percentage displacement errors measured in mono-beam mode based on data averaged over ten pulses at photon energies of (*a*) 500 eV, (*b*) 700 eV, (*c*) 900 eV and (*d*) 1100 eV for different voltage configurations: Set A, original configuration; Set B, electrodes 1 and 2 floating; Set C, simulation-derived configuration with near-unity magnification. Compared with pink-beam operation, saturation effects are reduced, allowing a more reliable comparison of voltage-configuration dependence. No single voltage configuration consistently outperforms the others across all photon energies. The results are based on the average of every ten pulses.

Mono-beam operation, in contrast, substantially reduced the influence of saturation and enabled a more meaningful evaluation of voltage-configuration dependence. Fig. 8 ▸ summarizes the relative percentage displacement errors obtained from data averaged over ten pulses in mono-beam mode at photon energies of 500, 700, 900 and 1100 eV, for various combinations of repeller voltage and MCP gain, with the effective magnification taken into account.

At 500 eV and 700 eV, weak saturation-like behavior was still observed, particularly at higher MCP gain settings. Under these conditions, the displacement error was generally smaller when the MCP gain voltage was set to −1400 V compared with higher gain values, consistent with a reduced saturation. Despite this residual nonlinearity, the error values were sufficiently stable to permit a qualitative comparison among voltage configurations.

At 900 eV and 1100 eV, the relative displacement error was typically below approximately 2% for most voltage configurations, except when the repeller voltage was set to 2000 V. Once saturation effects were sufficiently suppressed, the beam position measurement accuracy showed limited sensitivity to the specific electrode voltage configuration. As indicated in Fig. 8 ▸, no single configuration (Set A, Set B or Set C) consistently provided superior performance across the full photon-energy range.

Although the Set C configurations were designed to achieve an effective magnification close to unity based on electrostatic simulations, their displacement accuracy was not systematically better than that of the original voltage configuration. These observations suggest that, under mono-beam conditions with controlled signal levels, the improved XIBPM provides stable beam position measurements that are relatively insensitive to moderate variations in electrode voltages, offering practical flexibility for beamline operation.

#### 4.3.3. Beam position resolution

In addition to the relative displacement error discussed above, the beam position resolution of the XIBPM was evaluated under mono-beam operation, where saturation effects are minimized and signal conditions remain stable. The resolution was determined by analyzing the statistical fluctuation of the extracted beam position using both pulse-by-pulse data and profiles averaged over ten pulses.

For a fixed voltage configuration, the beam position was obtained from the XIBPM profile by identifying the pixel corresponding to the maximum signal intensity for each recorded pulse. At each mirror position, the standard deviation of the peak pixel position was calculated from the pulse-by-pulse data. The same procedure was applied to the peak positions extracted from profiles averaged over ten pulses. The beam position resolution was then defined as the average of these standard deviations over all investigated mirror positions for a given photon energy.

The resolution was first evaluated separately for the January and May 2025 machine-study campaigns. In the January measurements, the beam position resolution was 28.1 ± 13.8 µm for pulse-by-pulse analysis and 11.1 ± 5.3 µm for data averaged over ten pulses. In the May campaign, corresponding values of 21.5 ± 8.4 µm (pulse-by-pulse) and 8.7 ± 6.0 µm (averaged over ten pulses) were obtained. Although conducted during separate machine-study periods, the results from January and May are consistent within their respective uncertainties. In both cases, a clear improvement in resolution is observed when moderate pulse averaging is applied.

To obtain a representative estimate of the achievable beam position resolution, the January and May data sets were combined. The combined analysis yields an average resolution of approximately 24–25 µm for pulse-by-pulse measurements, improving to approximately 10–12 µm when averaging over ten pulses.

These results indicate that the beam position resolution of the XIBPM can be significantly improved through moderate pulse averaging. In routine beamline operation, position monitoring is typically based on averaged signals rather than shot-to-shot data. Under these conditions, a resolution on the order of 10 µm can be achieved with averaging over ten pulses, which is adequate for stable beamline diagnostics.

To quantify the improvement achieved by the redesign, the beam position resolution of the original XIBPM configuration was also evaluated using the same analysis procedure. The measurements before the redesign were also performed in Kr gas mode under mono-beam operation. The available data before the redesign were obtained at an MCP gain voltage of −1400 V, whereas corresponding data at −1600 V were not available. Under this condition, the original configuration yielded resolutions of 38.0 ± 26.2 µm for pulse-by-pulse analysis and 17.4 ± 12.4 µm for data averaged over ten pulses. Compared with the representative values obtained after the redesign, this corresponds to an overall improvement of approximately 36% in beam position resolution.

#### 4.3.4. Profile characteristics

Despite the improved stability and accuracy of beam position measurements achieved through the internal structure redesign, the transverse beam profiles measured by the XIBPM remain substantially broader than the photon beam sizes determined by independent diagnostics. This behavior was consistently observed over the investigated photon energy range and under various operating conditions.

Quantitative comparisons with wire-scan measurements show that the FWHM of the XIBPM profiles is approximately one order of magnitude larger than the corresponding photon beam size. Although the measured profile width follows the general energy dependence of the photon beam size, its absolute scale is largely governed by the transverse spread of photo-ions during transport. This broadening arises from the initial thermal velocity and angular distribution of the ions, as well as from the intrinsic point-spread function of the MCP–phosphor readout system.

Profile widths obtained under different internal configurations were also examined. For photon energies affected by saturation effects, the measured widths are not reliable and are therefore excluded from the comparison. Focusing on the unsaturated case at 1100 eV, the profile widths measured during the January campaign with the mesh-based electrode structure were found to be comparable with those obtained during the May campaign after removal of the mesh electrodes. This observation indicates that the overall scale of the measured profile width is largely insensitive to the presence of the mesh electrodes.

A similar comparison was performed between pink-beam and mono-beam operation under the same internal configuration during the May campaign. Despite differences in spectral bandwidth and intensity characteristics, the XIBPM profile widths measured at 1100 eV were comparable for the two beam modes. This result suggests that the dominant contribution to the transverse profile broadening originates from ion transport and the readout characteristics rather than from intrinsic properties of the photon beam.

These observations confirm that the XIBPM is well suited for beam position monitoring based on the extracted peak position of the ion distribution. However, the measured profile width primarily reflects the response characteristics of the gas-ionization detection process and should not be interpreted as a direct measure of the transverse photon beam size.

## 5. Conclusions

An improved X-ray ionization beam position monitor for soft X-ray free-electron lasers has been developed and evaluated through dedicated machine-study campaigns at PAL-XFEL. The redesign was guided by electrostatic simulations and focused on mitigating internal electric-field distortions and enhancing the mechanical stability of the detector structure.

The introduction of a conductive shielding cover reduced electric-field distortion originating from the MCP high-voltage lead, resulting in improved displacement accuracy and enhanced measurement stability. Across the investigated photon-energy range, this modification consistently improved the reliability of beam position measurements and represents the most significant structural improvement identified in this study.

The mesh-free electrode structure reduced ion loss associated with the mesh electrodes and increased ion transmission, leading to enhanced signal amplitude and greater flexibility in voltage selection. However, its influence on position accuracy depended on operating conditions. Under high-intensity XFEL operation, particularly in pink-beam mode, the increased signal amplitude occasionally led to saturation effects.

Because soft X-ray FELs inherently provide high photon flux, sufficient signal-to-noise ratios can be achieved with the original mesh-based electrode structure under typical operating conditions. During beamline commissioning performed in December 2025, stable operation was achieved by implementing the shielding cover while retaining the mesh electrodes. This experience suggests that the shielding cover is the key modification for improving displacement accuracy, whereas the electrode configuration can be selected according to beam intensity and operational constraints.

Under optimized conditions, the improved XIBPM demonstrated reliable beam position monitoring over photon energies of 500–1100 eV in both vertical and horizontal configurations. A beam position resolution of approximately 24–25 µm was obtained for pulse-by-pulse measurement and improved to approximately 10–12 µm when averaging over ten pulses.

The transverse profiles measured by the XIBPM were substantially broader than the photon beam size determined by wire-scan measurements. This broadening is primarily attributed to transverse photo-ion transport and the intrinsic point-spread characteristics of the MCP–phosphor detection system. Accordingly, the XIBPM is best suited for beam position monitoring based on the extracted peak position of the ion distribution rather than for direct beam size characterization.

Overall, the improved XIBPM provides a practical and non-invasive solution for beam position diagnostics in soft X-ray FEL beamlines.
